# A Prospective Study on the Systematic Application of the Safe Insertion Umbilical Venous Catheter (SIUVeC) Bundle

**DOI:** 10.3390/children12070819

**Published:** 2025-06-21

**Authors:** Giovanni Barone, Gina Ancora, Mauro Pittiruti, Giorgia Prontera, Giovanni Vento, Vito D’Andrea

**Affiliations:** 1 Neonatal Intensive Care Unit, AUSL della Romagna, Ospedale Infermi, 47921 Rimini, Italy; gina.ancora@auslromagna.it; 2 Department of Surgery, Fondazione IRCSS Policlinico A. Gemelli, 00168 Rome, Italy; mauro.pittiruti@policlinicogemelli.it; 3 Neonatal Intensive Care Unit, Fondazione IRCSS Policlinico A. Gemelli, 00168 Rome, Italy; giorgia.prontera@libero.it (G.P.); giovanni.vento@policlinicogemelli.it (G.V.); vito.dandrea@policlinicogemelli.it (V.D.)

**Keywords:** umbilical venous catheter, neonatal intensive care unit, central venous catheter, preterm birth

## Abstract

**Background/Objectives**: Inserting umbilical venous catheters is a common procedure in neonatal intensive care units. However, this maneuver is potentially associated with early and late complications, some of which can be severe. Several strategies have been described in the literature to minimize the risk of such complications. The recently described SIUVeC (Safe Insertion Umbilical Venous Catheter) protocol incorporates all the innovations suggested by the latest literature, with the intention of reducing the risk associated with this procedure. The purpose of this paper is to report the outcomes of the systematic implementation of this protocol. **Methods**: Infants were enrolled in this prospective study if they were eligible for umbilical venous catheter placement. **Results**: A total of 449 infants were enrolled in the study. In total, 407 (90.6%) catheters were successfully placed in the proper position, at the inferior cavo–atrial junction. A total of 89.9% of the catheters were removed electively, without any complications. **Conclusions**: The SIUVeC protocol demonstrates an effective strategy for the safe elective insertion of umbilical venous catheters.

## 1. Introduction

The insertion of umbilical venous catheters (UVCs) is a widely used procedure in neonatal intensive care units (NICUs). These devices provide fast and high-performance venous access for critically ill neonates. However, UVC placement is also associated with significant risks, such as malposition, infection, thrombosis, and extravasation [1,2,3,4]. In the past decade, the incidence of UVC-related complications has decreased due to the implementation of several innovative strategies.

Among these strategies, the most significant has been the extensive use of ultrasound (US) for “tip navigation” (i.e., assessing the correct direction of the catheter as it advances into the vascular system) and “tip location” (i.e., ensuring that the catheter tip is correctly positioned, centrally) [5]. The use of US helps to reduce the risk of primary malposition [6], thereby decreasing the likelihood of associated complications [7].

Other evidence-based strategies, such as adherence to maximal barrier precautions, skin antisepsis with 2% chlorhexidine in alcohol, and the use of cyanoacrylate glue for securing the exit site, are also important factors in reducing complications [8,9,10].

Recently, these strategies have been consolidated into an “insertion bundle”, known as the SIUVeC protocol (Safe Insertion Umbilical Venous Catheter) [11]. It was designed by the Italian group, GAVePed, which is the pediatric interest sub-group of the most important Italian group on venous access. An “insertion bundle” is a simple list of clear recommendations based on the best available scientific evidence. These recommendations work synergistically to ensure maximum patient safety and to reduce the risk of procedure-related complications [12].

While each individual strategy within the SIUVeC bundle has been shown to be effective, no study has yet evaluated the combined application of all these strategies to achieve a greater overall effect. The aim of this paper is to report on the impact of systematically applying the SIUVeC bundle in a large cohort of newborns who were admitted to the NICU.

## 2. Materials and Methods

This was a multicenter prospective study, conducted in two large Level III NICUs (Infermi Hospital in Rimini and Fondazione Policlinico A. Gemelli in Rome). The study protocol was approved by the local Ethics Committee, with the number ID 3044. The guardians and parents were informed of the study objective and signed an informed consent form. All patients admitted to the NICUs and with an indication to place a UVC were eligible for the study. Only elective UVC insertions were considered. The indications for catheter placement were based on the Italian Consensus, developed by the Italian group GAVePed, known as the DAV–expert (vascular access device-expert) algorithm, which has been published elsewhere [13].

Therefore, the only exclusion criteria were as follows: the refusal of consent and the emergency insertion of a UVC in the delivery room.

The list of indication for UVC insertion, according to the DAV-expert [13], included the following:

(a) preterm infants born at or less than 28 weeks of gestation; (b) neonates with severe respiratory distress (intubated or on non-invasive ventilation with FiO_2_ > 40%), with hemodynamic instability or with difficulty in finding peripheral venous access at the time of birth; and (c) neonates affected by asphyxia, requiring therapeutic hypothermia.

This prospective study was designed to analyze the clinical impact of an evidence-based insertion bundle on the rate of any complication potentially related to central venous catheterization in a large population of neonates.

The eight-step bundle for minimizing insertion-related complications associated with UVCs, the “SIUVeC” protocol (Safe Insertion Umbilical Venous Catheter), consists of the following steps:

*(1)*  *Preprocedural Evaluation*

Ultrasound (US) plays a crucial role in this process, as it helps to identify anatomical variants, such as the right umbilical veins or inferior vena cava anomalies; evaluate the patency of the ductus venosus, which can affect catheter placement; and assess the angle between the ductus venosus and portal sinus, which influences the risk of malposition [6]. Preprocedural ultrasounds can also aid in selecting the correct catheter size, reducing the risk of thrombosis by ensuring the catheter diameter is appropriate for the vein [14].

*(2)*  *Preassembled Insertion Kits*

The use of preassembled insertion kits is recommended to streamline the procedure and reduce infection risks. These kits should contain all the necessary sterile components for safe and efficient catheter insertion, and they should be readily available in NICUs. Guidelines from organizations such as the Society for Healthcare Epidemiology of America (SHEA) and the Infusion Nursing Society (INS) recommend the use of such kits to maintain aseptic conditions during the procedure [15,16].

*(3)*  *Appropriate aseptic techniques (hand hygiene, maximal barrier precautions, and skin antisepsis with 2% chlorhexidine in 70% isopropyl alcohol)*

Maintaining an appropriate aseptic technique is vital to preventing infections. Key elements include hand hygiene, typically using hydroalcoholic gel or soap and water.

Proper skin antisepsis using 2% chlorhexidine in 70% alcohol, applied gently to avoid chemical burns, particularly in premature infants [9].

Full maximal barrier precautions are also essential in reducing the risk of infective complications.

*(4)*  *Vein cannulation using the smallest catheter that meets the infusion requirements and choosing wisely between single- or double-lumen UVCs*

To prevent venous thrombosis, the external diameter of the catheter should not exceed one-third of the vein’s internal diameter [14]. In neonates, the optimal catheter size is not well established, but studies suggest that using the smallest catheter that meets the infant’s needs can reduce the thrombosis risk. Double-lumen catheters, which have a larger diameter, should be reserved for specific cases, such as frequent blood sampling or multiple non-compatible infusions.

*(5)*  *Real-time tip navigation and tip location by US (according to the NeoECHOTIP protocol [17])*

Real-time ultrasound has largely replaced traditional methods, such as X-ray, for guiding UVC insertion. US allows for both tip navigation and tip location, reducing the incidence of primary malposition (which can occur in up to 50% of the cases without ultrasound) [6,18]. By providing direct visualization, ultrasound improves catheter progression and reduces errors, ensuring the catheter tip is correctly positioned at the junction of the inferior vena cava and right atrium.

*(6)*  *Securement of the catheter and protection of the exit site (combining sutureless devices, cyanoacrylate glue, and semipermeable transparent membranes)*

The securement and protection of the UVC exit site are crucial in preventing complications such as dislodgement and infection. Cyanoacrylate glue has been shown to effectively secure the catheter, reducing early dislodgement; a multimodal strategy combining glue, sutureless devices, and transparent membranes may offer optimal protection [19]. In this protocol, 3–4 drops of octyl-butyl-cyanoacrylate glue are applied circumferentially around the catheter and onto Wharton’s jelly. After allowing a few seconds for the glue to dry, a transparent semipermeable membrane is applied to cover the site.

*(7)*  *Post-procedural serial assessment of tip location by US*

UVCs can experience secondary malposition or migration, especially after the initial insertion [20,21]. Regular post-procedural ultrasound assessments at 24–48 h and again at 72–96 h can help to detect and correct malposition early.

*(8)*  *Early removal of the device (within 4–5 days)*

The early removal of the UVC (preferably within 4–5 days) is recommended to reduce the risk of bloodstream infections, as the risk increases with the duration of catheter use [2,15,22].

All procedures were performed in the neonatal intensive care unit (NICU) by the attending neonatologists, who were fully trained for the adoption of the insertion bundle.

The full training program was published elsewhere [5] and included a formal classroom lesson of approximately 40 mins; bedside US practice after the theoretical training, showing how to set the US machine and teaching how to identify anatomic and vascular structures; bedside US teaching on how to perform a complete US-guided UVC placement; the distribution of informative teaching material; and a weekly follow-up survey. After insertion, the maintenance of all UVCs was carried out according to the hospitals’ policies (e.g., dressings changed only when soaked or detached, needle-free connectors with neutral displacement, disinfecting caps, flush and lock with saline only, and surveillance of the exit site).

The surveillance of the catheter exit site was conducted by the attending nurse at least once per shift. The use of a transparent, semipermeable membrane facilitated thorough visual inspections of the site. The parameters assessed included the condition of the skin beneath the dressing, with particular attention paid to signs of hyperemia and to the length of the external tract of the catheter to detect any potential dislodgement. All findings were documented in the medical records and any significant changes were promptly reported to the attending physician.

The endpoint was to evaluate the incidence of any immediate or late complications occurring during the procedure; the number of devices electively removed; and the reason for any early, unplanned removals. Data about the catheters were also recorded, such as their diameter, mean dwell time, and the number of lumens.

Catheter-related bloodstream infection (CRBSI) was defined according to the IDSA guidelines [23], using the differential time to positivity. This is characterized by the growth of the same microorganism from both the catheter and peripheral blood cultures, with the culture drawn from the catheter becoming positive at least 2 h earlier than the peripheral one [24].

If catheter dislodgement was detected during routine post-procedural ultrasound assessments or at the inspection of the external tract of the catheter, appropriate corrective measures were taken based on the direction of migration. In cases of inward migration, the catheter was repositioned by carefully pulling it back to the correct location under ultrasound guidance. If outward migration had occurred and the catheter tip was no longer properly positioned at the inferior cavo–atrial junction, the catheter was removed and replaced with a new vascular access device at the earliest appropriate opportunity

## 3. Results

The study was conducted between January 2021 and December 2024. In total, 449 neonates who were admitted to the NICUs were eligible for UVC placement, according to the DAV-expert consensus [13]. All catheters were inserted according to the SIUVeC protocol [11]. The baseline characteristics of the enrolled population are summarized in Table 1. The continuous variables are expressed as mean ± standard deviation (min–max) since the distribution of the data was normal, according to the D’agostino–Pearson test. The reasons for UVC insertion and the catheter characteristics are summarized in Table 2.

The number of catheters placed in the proper position (the inferior cavo–atrial junction) was 407 (90.6%). All 42 low-lying catheters (9.3%) were electively removed within 24 h and a different vascular access device was subsequently placed. Among the 407 catheters placed in the proper position, 366 were electively removed (89.9%); 3 of the 407 (0.7%) were removed as a consequence of the diagnosis of catheter-related bloodstream infection; 10 (2.4%) catheters caused the development of thrombosis within the umbilical vein and/or the ductus venosus and were subsequently removed without any specific treatment; and 28 catheters were removed as a consequence of secondary malposition (6.8%). In total, 12 catheters were found to have migrated inward towards the heart during US assessments and were pulled back in the proper position under US guidance. Data about the UVCs are summarized in Table 3.

## 4. Discussion

**Figure 1:**
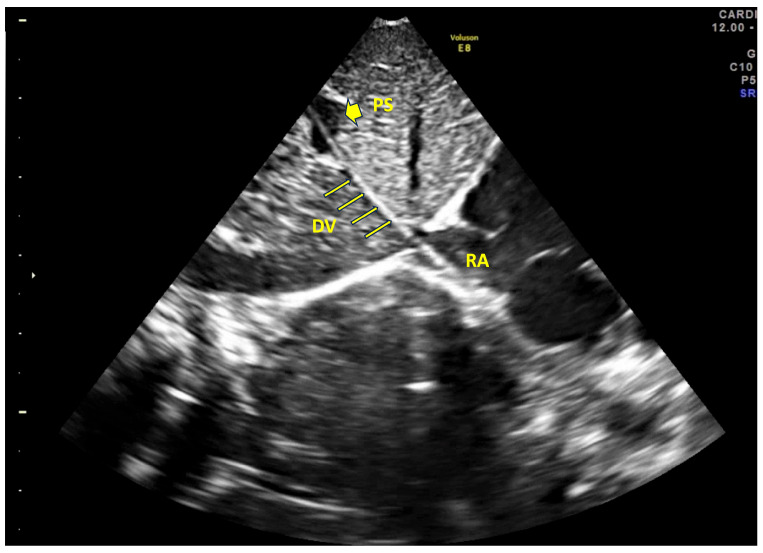
Ultrasound image. In this acoustic window, the portal sinus (PS), ductus venosus (DV), and right atrium (RA) are clearly aligned, facilitating the correct advancement of the catheter. The catheter is visible along its course, with the tip located in the right atrium.

Umbilical venous catheters represent a widely adopted strategy for providing central venous access in critically ill or preterm neonates requiring resuscitation, intravenous medications, and/or parenteral nutrition. Despite their clinical utility and relatively straightforward insertion, UVCs are associated with a significant risk of both infectious and non-infectious complications [1,25,26]. Our prospective study, which was conducted over a four-year period and included 449 neonates, provided insights into the outcomes associated with UVC use when inserted and managed under a standardized protocol (SIUVeC), aligned with expert consensus recommendations. A key finding of our study was the high rate of correct UVC placement, with 407 catheters (90.6%) positioned at the inferior cavo–atrial junction, the optimal site recommended by international guidelines [15]. This is a noteworthy achievement, as previous reports have documented lower success rates (approximately 50%), especially in the absence of dedicated protocols or real-time ultrasound guidance [18,27]. The use of the SIUVeC protocol, which includes the point-of-care ultrasound (POCUS) protocol, NeoECHOTIP, for tip navigation and tip location, is the main reason for this significant outcome (see Figure 1).

All 42 catheters (9.3%) that were found to be low-lying (i.e., within the portal sinus or the umbilical vein) were removed electively within 24 h and replaced by an alternative vascular access. This prompt management of malpositioned catheters most likely prevented the onset of serious complications, such as hepatic injury or portal vein thrombosis, both of which have been described extensively in the literature [28,29,30].

**Figure 2:**
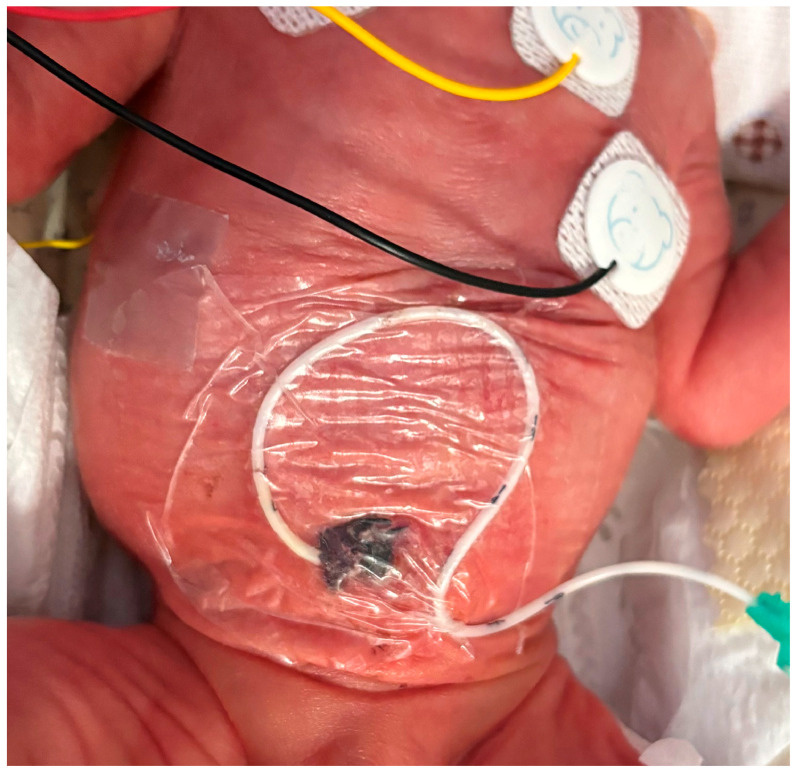
Securement of UVC using cyanoacrylate glue and semipermeable transparent membrane.

Catheter migration is a well-documented phenomenon, occurring in up to 50–60% of cases, depending on the study design and monitoring methods [18,19,20]. In our cohort, only 28 catheters (6.8%) were removed due to secondary malposition. This significant result was most likely due to the novel securement, which included the systematic use of cyanoacrylate glue and a semipermeable, transparent membrane to cover the whole exit site (including Wharton’s jelly, see Figure 2). However, considering the high risk of severe consequences resulting from a malpositioned catheter, it is very important to double-check the catheter tip using US in a timely manner, as proposed by the SIUVeC protocol.

Catheter-related bloodstream infections (CRBSIs) are among the most severe complications associated with central lines, particularly in preterm neonates, where they are linked to increased morbidity, including adverse neurodevelopmental and growth outcomes [12,25,31]. In our study, only three catheters (0.7%) were removed due to CRBSI. This incidence is considerably lower than the reported rates of 3–20% in the literature [32] and may reflect the impact of the strict adherence to aseptic techniques; the use of line care bundles; and the new securement technique, which might have had an impact on the extraluminal colonization.

Among the non-infectious complications, thrombosis is one of the most commonly reported. In our cohort, 10 UVCs (2.4%) were removed due to thrombosis involving the umbilical vein and/or ductus venosus. This incidence is consistent with previous studies, with reporting rates ranging from 2.2% to over 40% [29], with the variability being largely attributed to differing surveillance practices and imaging protocols. No cases of hepatic complications, such as extravasation or hepatic parenchymal injury, were reported in our dataset. This is probably related to the early removal of low-lying UVCs and the high rate of properly located UVCs.

The major limitation of the present study was the absence of a control group. However, all the observed outcomes were more favorable than those commonly reported in the literature, suggesting that conducting a randomized controlled trial may raise ethical concerns related to clinical equipoise.

**What** **is already known**

• Umbilical venous catheters (UVCs) are widely used in NICUs to provide central venous access, but are associated with a high risk of complications, including malposition, infection, and thrombosis.
• Proper tip location using US and securement strategies are essential in minimizing these risks.

**What this study adds**

• This prospective study demonstrates that the implementation of a structured protocol (SIUVeC), which includes real-time ultrasound (NeoECHOTIP) and cyanoacrylate glue for securement, results in high rates of optimal tip placement (90.6%) and low complication rates.
• This study has significantly expanded the body of evidence supporting the systematic use of US before, during, and after UVC insertion to enhance both the safety and efficacy of the procedure. The consistent application of the DAV-expert algorithm and the SIUVeC protocol proved to be valuable tools in minimizing the risks traditionally associated with UVC use in the NICU setting.

## 5. Conclusions

The favorable outcomes observed in our study support the use of a structured, evidence-based protocol for UVC insertion and maintenance. The SIUVeC protocol, combined with the routine NeoECHOTIP protocol and a proactive catheter management strategy, led to high rates of optimal placement and low complication rates. Nonetheless, the decision to place a UVC must be carefully weighed against the potential complications. When used, UVCs should be closely monitored, reassessed frequently, and removed as soon as clinically feasible. These strategies are essential in improving safety and outcomes for the most vulnerable neonatal population.

## Figures and Tables

**Table 1:** Baseline characteristics of enrolled infants. Data are expressed as mean + standard deviation or as a number (percentage).

	Mean (SD)/Number (%)	Min–Max
Gestational age	32.7 ± 5.3	22–41.6
Birth weight	1922 ± 1098	350–4700
Male	230 (51.2%)	
Small for gestational age	123 (27.3%)	

**Table 2:** Reason for umbilical venous catheter insertion and catheter characteristics.

	Number (%)
Prematurity	176 (39.2%)
Severe respiratory distress syndrome	95 (21.2%)
Hemodynamic instability	87 (19.4%)
Difficult peripheral venous cannulation	41 (9.1%)
Therapeutic hypothermia	50 (11.1%)
Single-lumen catheter	177 (39.4)
Double-lumen catheter	272 (60.6%)
3.5 Fr catheter	145 (32.3%)
4 Fr catheter	258 (57.5%)
5 Fr catheter	46 (10.2%)

**Table 3:** Main outcomes. Data are expressed as mean + standard deviation or as a number (percentage). CRBSI—Catheter-related blood stream infection.

	Mean (SD)/Number (%)	Min–Max
Mean dwell time (days)	4.2 ± 2	1–7
No catheters in a safe position	407/449 (90.6%)	
Electively removed	366/407 (89.9%)	
CRBSI	3/407 (0.7%)	
Thrombosis	10/407 (2.4%)	
Secondary malposition	28/407 (6.8%)	

## Data Availability

The raw data supporting the conclusions of this article will be made available by the authors on request due to restrictions (legal and ethical reasons).
